# Mast Cells Play No Role in the Pathogenesis of Postoperative Ileus Induced by Intestinal Manipulation

**DOI:** 10.1371/journal.pone.0085304

**Published:** 2014-01-09

**Authors:** Pedro J. Gomez-Pinilla, Giovanna Farro, Martina Di Giovangiulio, Nathalie Stakenborg, Andrea Némethova, Annick de Vries, Adrian Liston, Thorsten B. Feyerabend, Hans-Reimwer Rodewald, Guy E. Boeckxstaens, Gianluca Matteoli

**Affiliations:** 1 Department of Clinical and Experimental Medicine, Translational Research Center for Gastrointestinal Disorders (TARGID), KU Leuven, Leuven, Belgium; 2 Department of Microbiology and Immunology, KU Leuven, Leuven, Belgium; 3 Autoimmune Genetics Laboratory, Vlaams Instituut voor Biotechnologie (VIB), Leuven, Belgium; 4 Division for Cellular Immunology, German Cancer Research Center (DKFZ), Heidelberg, Germany; National Cancer Institute, United States of America

## Abstract

**Introduction:**

Intestinal manipulation (IM) during abdominal surgery results in intestinal inflammation leading to hypomotility or ileus. Mast cell activation is thought to play a crucial role in the pathophysiology of postoperative ileus (POI). However, this conclusion was mainly drawn using mast cell-deficient mouse models with abnormal Kit signaling. These mice also lack interstitial cells of Cajal (ICC) resulting in aberrant gastrointestinal motility even prior to surgery, compromising their use as model to study POI. To avoid these experimental weaknesses we took advantage of a newly developed knock-in mouse model, *Cpa3^Cre/+^*, devoid of mast cells but with intact Kit signaling.

**Design:**

The role of mast cells in the development of POI and intestinal inflammation was evaluated assessing gastrointestinal transit and muscularis externa inflammation after IM in two strains of mice lacking mast cells, i.e. *Kit^W-sh/W-sh^* and *Cpa3^Cre/+^* mice, and by use of the mast cell stabilizer cromolyn.

**Results:**

*Kit^W-sh/W-sh^* mice lack ICC networks and already revealed significantly delayed gastrointestinal transit even before surgery. IM did not further delay intestinal transit, but induced infiltration of myeloperoxidase positive cells, expression of inflammatory cytokines and recruitment of monocytes and neutrophils into the muscularis externa. On the contrary, *Cpa3^Cre/+^* mice have a normal network of ICC and normal gastrointestinal. Surprisingly, IM in *Cpa3^Cre/+^* mice caused delay in gut motility and intestinal inflammation as in wild type littermates mice (*Cpa3^+/+^*). Furthermore, treatment with the mast cell inhibitor cromolyn resulted in an inhibition of mast cells without preventing POI.

**Conclusions:**

Here, we confirm that IM induced mast cell degranulation. However, our data demonstrate that mast cells are not required for the pathogenesis of POI in mice. Although there might be species differences between mouse and human, our results argue against mast cell inhibitors as a therapeutic approach to shorten POI.

## Introduction

Open abdominal surgery leads to impaired motility of the entire gastrointestinal (GI) tract, a condition referred to as postoperative ileus (POI) [1]. Depending on the type of surgery, POI may last several days and, in up to 10% of patients, it might be prolonged to over 2 weeks, with symptoms including nausea, vomiting, intolerance to food and absence of defecation [2]. In addition to significant patient morbidity, POI is associated with increased hospital costs [3]. Therefore, any reduction in the occurrence or duration of POI could lead to a significant reduction in hospitalization and related costs.

POI is an immune-mediated condition characterized by a localized inflammatory reaction in the muscularis externa evoked by intestinal handling during surgery [1], [4]. Macrophages residing in the muscularis externa and mast cells have been proposed to be the key players in this inflammatory cascade [1], [4]. Pharmacological or genetic (op/op mice) depletion of resident macrophages indeed resulted in a decrease of inflammatory mediators and diminished the recruitment of leucocytes into the muscularis supporting a role for intestinal macrophages in the induction of POI [5]. Activation of muscularis intestinal resident macrophages subsequently leads to cytokine and chemokine release, followed by an influx of leucocytes starting approximately 3–4 h after surgery [6]. Finally, incoming leucocytes in conjunction with muscularis macrophages (after surgery-related-IM) induce the synthesis of prostaglandins and nitric oxide that directly impair smooth muscle contractility and consequently lead to POI [7], [8].

In addition to activation of muscularis macrophages after abdominal surgery, one of the earliest observations in rodents and humans is the activation of peritoneal mast cells with the release of their mediators into the peritoneal cavity [9], [10]. The importance of mast cells in the inflammatory cascade triggered by IM was also suggested by experiments using mast cell stabilizers such as ketotifen and doxantrazole. The treatment with the above mentioned stabilizers reduced the inflammatory response and the delay in gastrointestinal transit 24 h after abdominal surgery. Moreover, mutant mice lacking mast cells (*Kit^W/Wv^* and *Kit^W-sh/W-sh^*) showed reduced intestinal infiltrate following IM while reconstitution with wild type mast cells restored the phenotype [9], [11]. However, the role of mast cells in POI is not free of criticism since the mediators measured (proteases and tryptases) can be released even by other immune cells, while the mast cell degranulation agonist (compound 48/80) and stabilizers (ketotifen and doxantrazole) used are not specific for mast cells. In addition to the specificity of the compounds tested, the use of mast cells deficient mice based on Kit mutations is ambiguous, as the strains used (*Kit^W/Wv^* and *Kit^W-sh/W-sh^*) have alterations in multiple cell types of both immune and non-immune origin in addition to the mast cell defect [12]–[17]. In particular, Kit is necessary for the development of interstitial cells of Cajal (ICC), with both *Kit^W/Wv^* and *Kit^W-sh/W-sh^* strains having severe alteration of the ICC networks in the intestinal wall [15]–[17], and thus these mutations may cause mast cell-independent defects in gut motility.

To avoid this experimental bias in the current study, we used a genetic modified mouse strain with a targeted insertion of Cre-recombinase into the Carboxypeptidase A3 (*Cpa3*) locus (*Cpa3^Cre/+^* mice). This intervention leads to the specific mast cell ablation in tissues by a genotoxic transformation related protein 53 (Trp53)-dependent mechanism [18], [19]. In contrast to Kit mutants, *Cpa3^Cre/+^* mutants have a selective mast cell depletion and apart from a reduction in basophil numbers, other subpopulations of immune cells are intact [18]. Therefore, this new transgenic mouse model gives us the opportunity to specifically evaluate the role of mast cells in POI.

Here we show that *Kit^W-sh/W-sh^* mice have impaired gut motility at baseline due to the alterations on ICCs distribution, making this mouse strain unsuitable to study the role of mast cells in POI. By contrast, the selective depletion of mast cells (and partially of basophils) does not affect GI motility and does not prevent the development of IM-induced muscular inflammation and POI. Taken together, our data indicate that mast cells are not crucial in the development of POI.

## Materials and Methods

### Animals

Wild type mice (C57BL/6JOlaHsd; *WT*) were purchased from Harlan. *Kit^W-sh/W-sh^* mice were obtained by homozygote mating of mice originally purchased from The Jackson Laboratory [20]. *Cpa3^Cre/+^* gene-targeted mice have been described previously [18], [21]. Mice were kept at the KU Leuven animal facility under SPF conditions. All experimental procedures were approved by the Animal Care and Animal Experiments Committee of the Medical Faculty of the KU Leuven (Leuven, Belgium).

### Surgical procedure to induce postoperative ileus

Mice were anesthetized by intraperitoneal injection (i.p.) of a mixture of Ketamine (Ketalar 100 mg/kg; Pfizer) and Xylazine (Rompun 10 mg/kg; Bayer). Anesthetized mice underwent a laparotomy alone or a laparotomy followed by IM [9], [22]–[24]. Surgery was performed using a sterile moist cotton applicator attached to a device enabling the application of a constant pressure of 90 mN [25]. The small intestine was manipulated three times from the caecum to the distal duodenum. During and after the surgical procedure, mice were positioned on a heating pad (32°C) until they recovered from anesthesia. No pharmacological treatment was used to avoid influence on the outcome of the study.

### Gastrointestinal transit measurements

To assess GI transit, 10 µl of a liquid non-absorbable fluorescein isothiocyanate-labeled dextran (FITC-dextran, 70,000 Da; Invitrogen) was administered intragastrically 24 hours postoperatively to fasted animals. Ninety minutes after oral gavage, animals were sacrificed by CO2 overdose and the contents of stomach, small bowel (divided into 10 segments of equal length), caecum, and colon (divided in 3 segments of equal length) were collected and the amount of FITC in each bowel segment was quantified using a spectrofluorimeter (Ascent, Labsystem) at 488 nm. The distribution of the fluorescent dextran along the GI tract was determined by calculating the geometric center (GC): Σ (percent of total fluorescent signal in each segment x the segment number)/100 for quantitative comparison among experimental groups [5].

### Whole mount preparation and MPO staining

Twenty four hours after surgery, mice were sacrificed by CO2 overdose. The jejunum was quickly excised, and the mesenteric attachment removed. Jejunum segments were cut open along the mesentery border, fecal content was washed out in ice-cold modified Krebs solution, and segments were fixed with 100% ethanol for 10 minutes. Next, the mucosa and submucosa were removed and the remaining full-thickness sheets of muscularis externa were stained with Hanker Yates reagent (Sigma-Aldrich) for 10 minutes [26]. Myeloperoxidase (MPO) positive cells were visualized with a microscope (BX 41, Olympus) connected to a camera (XM10, Olympus). The number of MPO-positive cells was counted by an observer blind to the experimental conditions in 10 randomly chosen representative low-power magnification fields (acquired with the 10X objective, 668.4 µm x 891.2 µm).

### Staining and immunolabeling of mast cells, ICCs and intestinal muscularis macrophages

Mesenteric windows were carefully preserved and pinned down in a sylgard dish and subsequently fixed with 4% paraformaldehyde (Sigma-Aldrich) in PBS at 4°C for 10 minutes. To stain mast cells, mesenteric windows were incubated with 0.1% of toluidine blue (Sigma-Aldrich) for 1 hour and washed in PBS 3 times for 5 minutes.

Jejunum fragments were fixed with 4% paraformaldehyde and frozen in optimal cutting temperature compound (OCT; Neg 50; Thermo Scientific). Jejunum tissues were cut in 10-µm-thick transversal sections. After blocking for 2 hours at room temperature in 1% bovine serum albumin (BSA; Sigma-Aldrich) in PBS, sections were incubated with the primary antibodies rat anti Kit (clone 2B8; eBioscience) and rabbit anti Ano1 (Abcam) at a concentration of 1∶500 in 0.3% (vol/vol) Triton X-100 plus 1% BSA in PBS overnight at 4°C. Subsequently, sections were incubated with the appropriate secondary antibodies donkey anti-rat conjugated with CY5 (Jackson ImmunoResearch) and donkey anti-rabbit conjugated with CY3 (Jackson ImmunoResearch) at a concentration of 1∶1000 in 0.3% (vol/vol) Triton X-100 plus 1% BSA in PBS for one hour at room temperature. Sections were then counterstained with 4′,6-diamidino-2-phenylindole dilactate (DAPI; Invitrogen) to label nuclei.

Immunolabeling of intestinal resident macrophages was performed as follows. Jejunum muscularis externa whole mount preparations from naïve animals were subjected to two hours incubation with 1% bovine serum albumin (BSA, Sigma-Aldrich, St. Louis, MO) at room temperature (RT). After blocking, the preparations were incubated overnight with the primary antibody rat anti-F4/80 (1∶500, Biolegend) in PBS containing 1% BSA and 0.3% Triton X-100. The next day, the tissues were incubated for 1 hour at room temperature with the secondary antibody donkey anti-rat CY5 conjugated (1.1000, Jackson ImmunoResearch). Immunolabeled tissues were examined with an Olympus BX4 epifluorescence microscope (Olympus). Contrast and brightness of the pictures were adjusted using Image J software 1.46.

### Quantification of mouse mast cell protease-1 in peritoneal lavage fluid

Peritoneal lavage fluid was collected 30 minutes after IM by injection of 1 ml of warm sterile saline solution and a gentle massage of the peritoneum for 30 seconds. After that, peritoneal lavage fluid was collected and centrifuged at 300 g for 5 minutes at 4°C. The pellet was discarded and supernatant stored at −80°C until use. Peritoneal levels of mouse mast cell protease-1 (mMCP-1) as a measure of mast cell degranulation were measured by using a commercially available ELISA kit (eBioscience) following manufacturer's instructions. mMCP-1 levels were normalized to the protein concentration in the peritoneal lavage fluid.

### RNA extraction and inflammatory gene expression

Total RNA was extracted from the muscularis externa of the jejunum 24 hours after surgery. To this extent, tissue was homogenized by the TissueLyser II homogenizer (Qiagen). RNA extraction was performed using RNeasy Mini Kit (Qiagen) following the manufacturer's instructions. Total RNA was transcribed into complementary DNA (cDNA) by qScript cDNA SuperMix (Quanta Biosciences) according to the manufacturer's instructions. Quantitative real-time transcription polymerase chain reactions (RT-PCR) were performed with the LightCycler 480 SYBR Green I Master (Roche) on the Light Cycler 480, (Roche). Results were quantified using the 2-ΔΔCT method [27]. The expression levels of the genes of interest were normalized to the expression levels of the reference gene rpl32. PCR experiments were performed in triplicate. Primer sequences used are listed in Table S1.

### Cell isolation from the intestinal muscularis for flow cytometry

Twenty-four hours after the surgery, muscularis externa from the small intestine was isolated and enzymatically digested in MEMα medium (Lonza) containing 100 µg/ml of Penicillin, 100 µg/ml of Streptomycin, 50 µM β-mercaptoethanol, 5% FCS, 5 mg/ml protease type I (Sigma-Aldrich), 20 mg/ml collagenase type II (Sigma-Aldrich) and 5 U/ml DNase I for 15 min at 37°C. Cell suspensions were pre-incubated with an anti-FcR antibody (clone 24G2; BD Biosciences) and then stained with the following antibodies: CD45-APC-eFluor780 (30.F11, eBioscience), CD11b-PE-Cy7 (M1/70, BD Biosciences), CD64-Alexa Fluor647 (X54-5/7.1, BD Biosciences), Ly6G-PercPCy5.5 (IA8, BD Biosciences) and Ly6C-PE (AL-21, BD Biosciences). Stained samples were analyzed on a BD FACSCanto™ flow cytometer (BD Biosciences), and data were processed using FlowJo software (version 10.0.6, Tree Star). Cell numbers were calculated from flow cytometry frequencies using hemocytometer counts of trypan blue–excluding cells.

### Cromolyn administration

Mice received cromolyn (Disodium cromoglycate, Nalcrom®, Italchimici) by intraperitoneal injection of 30 mg/kg (in sterile saline solution) every 12 hours. The animals received cromolyn at three time points; 13 h and 1 h before IM and 11 h after IM (n = 10). Another group of mice (n = 10) received 200 µl of sterile solution (vehicle) at the same time points than cromolyn treated group. The researcher performing the surgeries was blinded for the type of pharmacological treatment.

### Statistical analysis

To compare multiple groups, one-way analysis of variance (one-way ANOVA) followed by Bonferroni post-hoc test was performed. Probability level of p<0.05 was considered statistically significant. Results are shown as mean ± standard error of the mean (SEM). Graph Pad Prism V.5.01 software was used to perform statistical analysis and generate graphs.

## Results

### Intestinal manipulation induces peritoneal mast cell degranulation

To define if peritoneal mast cell degranulation was induced during IM, we performed toluidine staining and quantified mouse mast cell protease-1 (mMCP-1) release in the peritoneal cavity of *WT* and *Kit^W-sh/W-sh^* mice.

**Figure 1:**
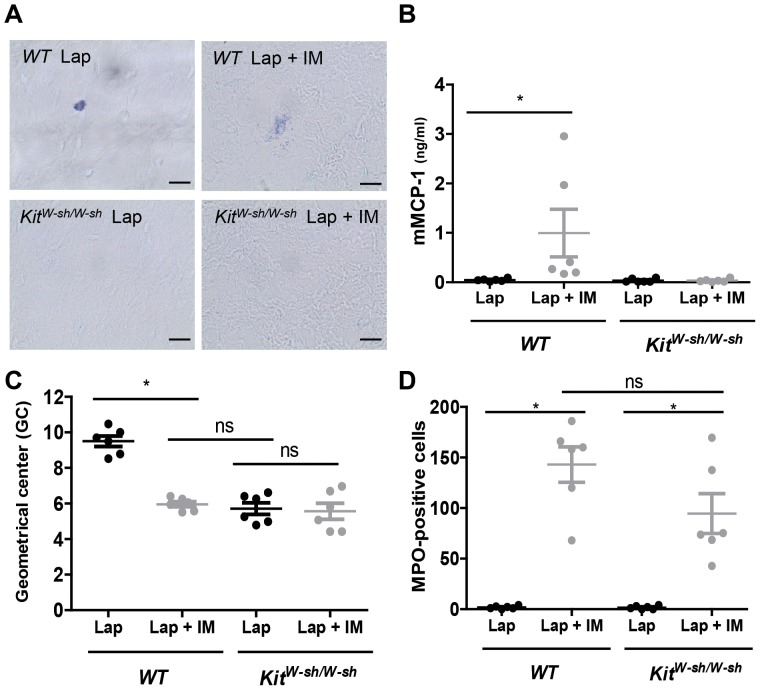
Intestinal manipulation in *Kit^W-sh/W-sh^* mice induces intestinal inflammation in the absence of mast cells. *WT* and *Kit^W-sh/W-sh^* mice were subjected to laparotomy alone (Lap) or to laparotomy plus intestinal manipulation (Lap + IM). (A) Mesenteric windows from *WT* and *Kit^W-sh/W-sh^* mutant mice were collected 30 min after surgeries and stained with 0.1% toluidine blue. Scale bar 50 µm. (B) 30 minutes after surgery the levels of mouse mast cell protease-1 (mMCP-1) was determined by ELISA in the peritoneal lavage fluid of *WT* and *Kit^W-sh/W-sh^*. (C) Geometric center (GC) values representing the dextran distribution through the GI tract 24 hours after surgery. (D) Infiltration of MPO-positive cells in the muscularis externa 24 hours after surgery. Data expressed as mean ± SEM. * P<0.01 (one-way ANOVA followed by Bonferroni post-hoc test). Dots represent individual mice.

Toluidine blue staining showed cells with typical dark-blue or purple cytoplasmic granules resembling mast cells in the mesenteric window of *WT* mice (Figure 1A). As expected, no mast cells were found in the mesenteric windows from *Kit^W-sh/W-sh^* mice (Figure 1A). IM induced typical signs of degranulation in *WT* mice, as visualized in Figure 1A by the appearance of dark-blue (toluidine blue-positive) structures released from and in the surrounding of a mast cell in the mesenteric window. In line, in the peritoneal lavage fluid of *WT* mice significant amount of mMCP-1 was detected already 30 minutes after IM (Lap; 0.036±0.0011 vs Lap +IM; 0.990±0.483 ng/ml; Figure 1B). As control, IM in the mast cell-deficient *Kit^W-sh/W-sh^* mutant mice did not lead to increase in peritoneal levels of mMCP-1 (Lap; 0.044±0.0013 vs Lap + IM; 0.039±0.009 ng/ml; Figure 1B).

As previously reported, IM in *WT* mice resulted in a significant delay in gastrointestinal transit (as measured by a reduction in the geometric center values, GC) compared to laparotomy (Figure 1C). In line with our previous observations IM led to recruitment of MPO-positive cells to the muscularis externa (Figure 1D). To define the role of mast cells in the pathogenesis of POI, IM was performed also in *Kit^W-sh/W-sh^* mutant mice. As shown in Figure 1C, gut transit was already significantly delayed in *Kit^W-sh/W-sh^* mutants undergoing laparotomy compared to control *WT* mice and IM did not worsen gastrointestinal transit in *Kit^W-sh/W-sh^* mice when compared to their laparotomy controls.

**Figure 2:**
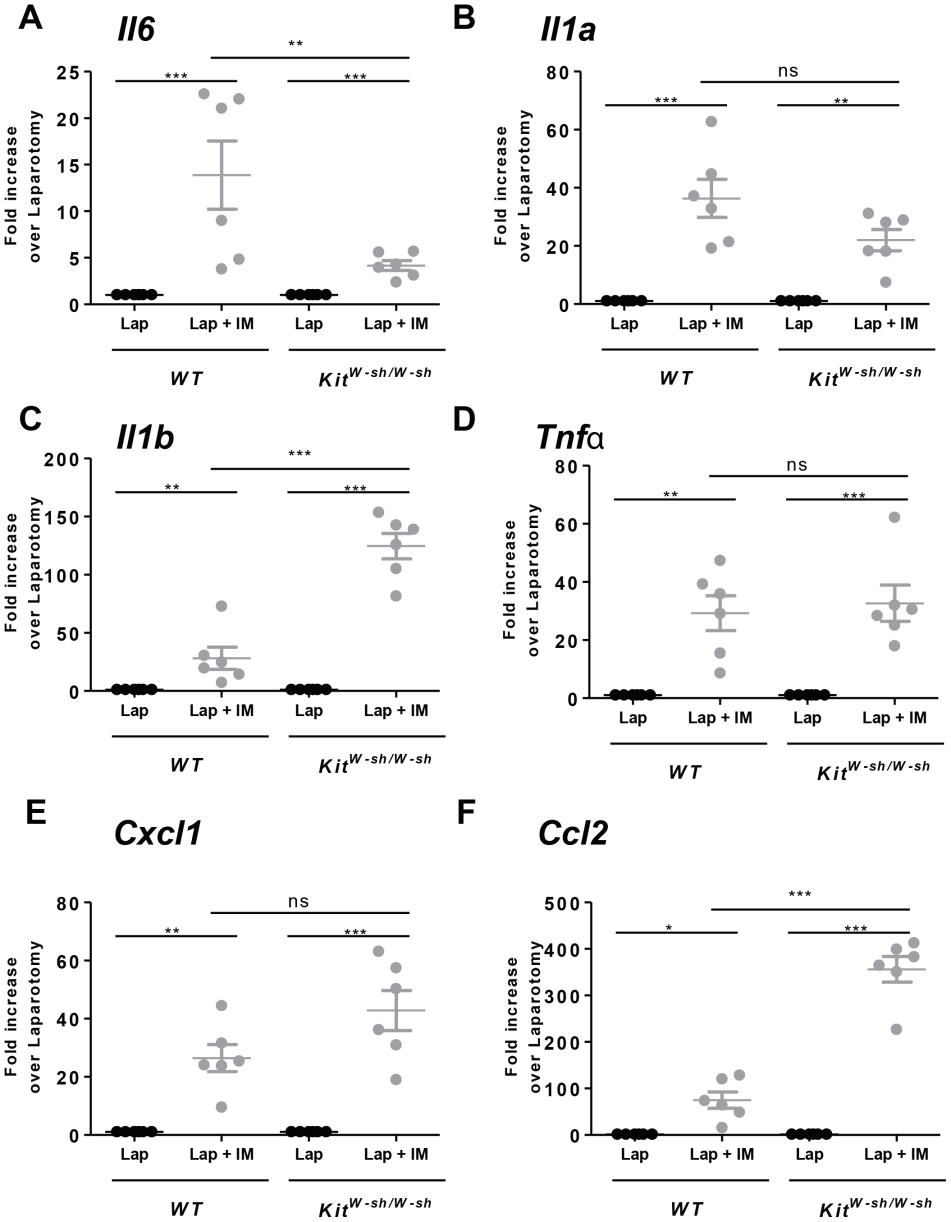
Intestinal manipulation in *Kit^W-sh/W-sh^* mice induces cytokines expression in the absence of mast cells. *WT* and *Kit^W-sh/W-sh^* mice were subjected to laparotomy alone (Lap) or to laparotomy plus IM (Lap + IM). Twenty four hours after surgery muscularis externa was collected and cytokines mRNA expression assessed by qPCR. *Il6* (A), *Il1a* (B), *Il1b* (C), *Tnfa* (D), *Cxcl1* (E) and *Ccl2* (F) mRNA expression was evaluated in the jejunum muscularis externa after 24 h. Data expressed as mean ± SEM. * P<0.05, ** P<0.01 or *** P<0.001 (one-way ANOVA followed by Bonferroni post-hoc test). Dots represent individual mice.

Interestingly, IM in *Kit^W-sh/W-sh^* mice resulted however in recruitment of MPO-positive cells to the muscularis externa with the same extent as in *WT* mice (*WT*; 143±18 number of cells per field vs *Kit^W-sh/W-sh^* 95±20 number of cells per field, ns, Figure 1D). Since IM induced recruitment of MPO-positive cells even in the absence of mast cells (*Kit^W-sh/W-sh^*) we analyzed the inflammatory response in the muscularis externa by assessing mRNA cytokine expression and the recruitment of immune cells. In line with the number of MPO-positive cells IM significantly increased cytokine mRNA expression (Il6, Il1a, Il1b, Tnfa, Cxcl1 and Ccl2; Figure 2) in the muscularis externa of *Kit^W-sh/W-sh^* mice when compared to laparotomy mice.

**Figure 3:**
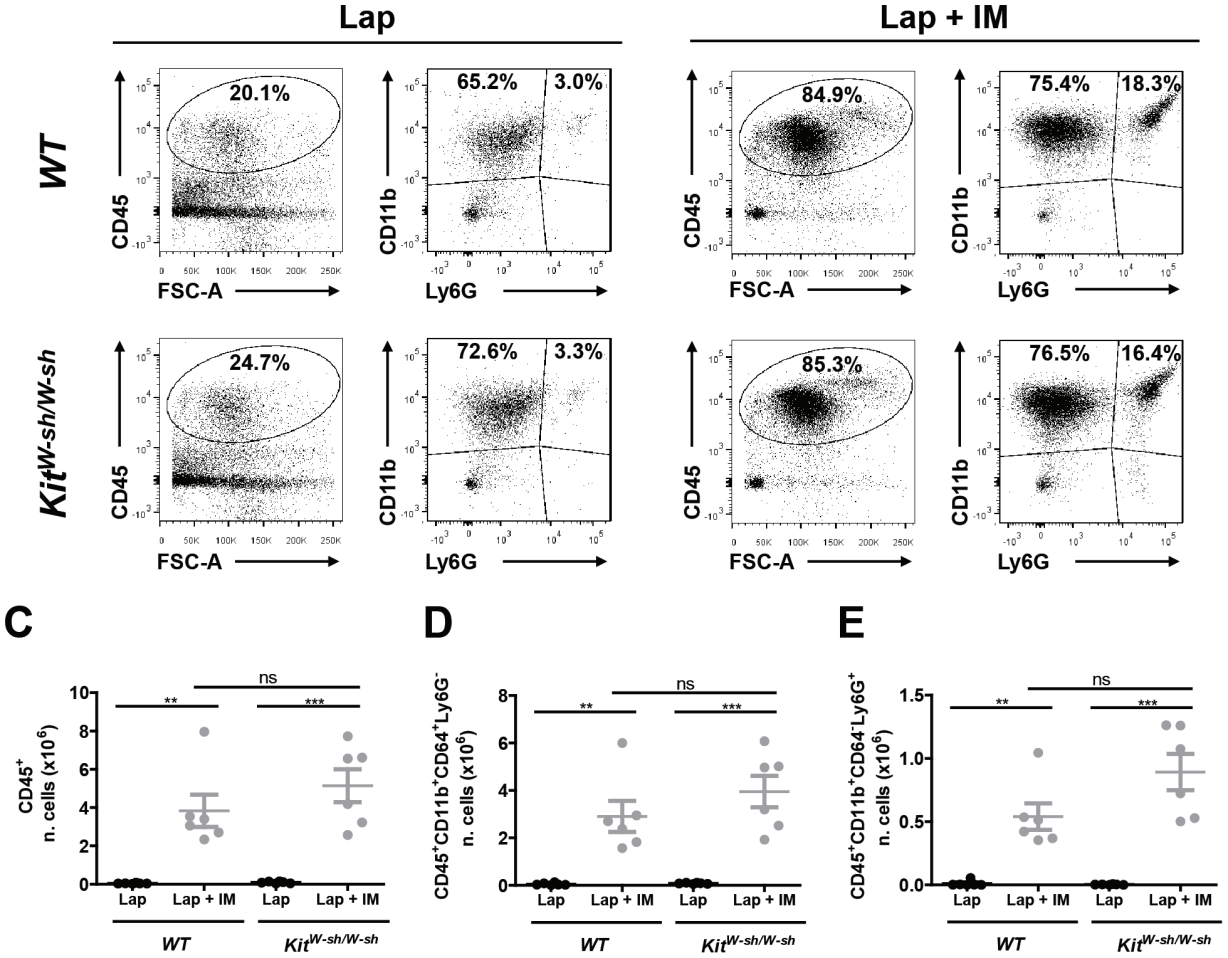
Intestinal manipulation in *Kit^W-sh/W-sh^* mice induces recruitment of immune cells in the muscularis externa in the absence of mast cells. *WT* and *Kit^W-sh/W-sh^* mice were subjected to laparotomy alone (Lap) or to laparotomy plus intestinal manipulation (Lap + IM) and immune cells recruitment in the muscle layer of the small intestine was assessed by flow cytometry. Typical dots plot showing different population of CD45-positive cells in *WT* and *Kit^W-sh/W-sh^* mice after (A) laparotomy or (B) laparotomy plus intestinal manipulation. Absolute number of CD45 positive immune cells (C), monocytes (D) and neutrophils (E) were calculated from flow cytometry frequencies using viable cell counts. Data expressed as mean ± SEM. ** P<0.01 or *** P<0.001 (one-way ANOVA followed by Bonferroni post-hoc test). Dots represent individual mice.

Intestinal manipulation-induced recruitment of immune cells to the muscularis is a typical event in POI. Therefore, we determined by flow cytometry the recruitment of immune cells in *WT* and *Kit^W-sh/W-sh^* mutant mice after laparotomy or laparotomy plus intestinal manipulation. As shown in Figure 3, IM induced a significant increase in CD45-positive immune cells, monocytes and neutrophils in the small intestine muscularis both in *WT* and *Kit^W-sh/W-sh^* mutant mice. Interestingly, no difference in the percentage and in the absolute number of monocytes and neutrophils were detected between *WT* and *Kit^W-sh/W-sh^* mutant mice (Figure 3C–D).

Taking into account that muscularis externa resident macrophages are suggested to be key players in the development of postoperative ileus in rodents and humans we performed F4/80 immunolabeling in jejunum whole mount muscularis preparations from *WT* and *Kit^W-sh/W-sh^* mutant mice. As shown in Figure S1 distribution and number of F4/80-positive resident macrophages are comparable in both *WT* and *Kit^W-sh/W-sh^* mutant mice.

### *Kit^W-sh/W-sh^* mice lack ICCs and have altered gastrointestinal transit

**Figure 4:**
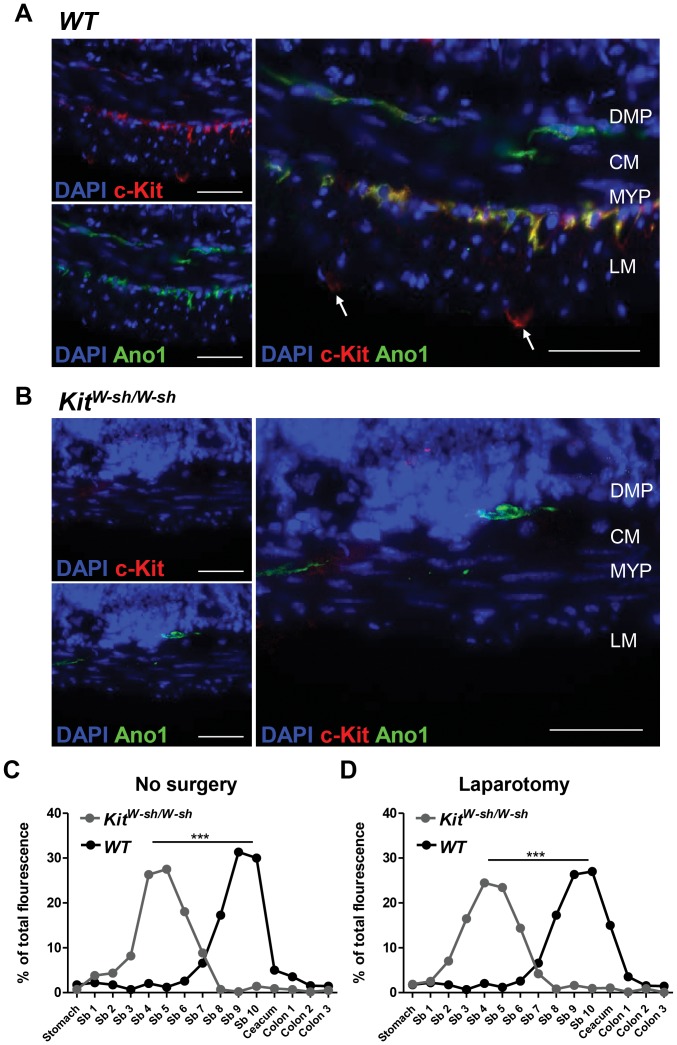
Deficient ICC network and intestinal dysmotility in *Kit^W-sh/W-sh^* mice. ICC network in the intestinal wall and GI transit were assessed in *WT* and *Kit^W-sh/W-sh^* mice. Jejunum sections from naïve *WT* mice (A) or *Kit^W-sh/W-sh^* mice (B) were immunolabeled with anti-Kit (red) and anti-Ano1 (green) antibodies. Sections were counterstained with DAPI (blue) to identify nuclei. White arrows are pointing Kit-positive and Ano1-negative mast cells in the jejunum from a *WT* mouse. DMP, deep muscular plexus; MYP, myenteric plexus; CM, circular muscle layer and LM, longitudinal muscle layer. Scale bar 50 µm. Ninety min after oral gavage with dextran-FITC naïve (C) or animals subjected to laparotomy (D) *WT* and *Kit^W-sh/W-sh^* were sacrificed and dextran-FITC distribution through the GI tract was determined as indicative of GI transit. Data expressed as mean. *** P<0.001 (two-way ANOVA).

Taking into account that Kit signaling is essential for the development of ICCs and that gut motility is impaired in *Kit^W-sh/W-sh^* mutant mice we performed immunolabelling of the ICC networks in *WT* and *Kit^W-sh/W-sh^* mice. As previously reported in the jejunum of *WT* mice, anti-Kit antibody labeled ICCs located at the level of the deep muscular and myenteric plexus regions of the muscularis externa (Figure 4A), as well as mast cells (white arrows Figure 4A, panel right). However, no Kit positive cells, mast cells or ICCs, were found in the muscularis externa from *Kit^W-sh/W-sh^* mutant mice (Figure 4B). To search for the presence of ICCs independently of Kit staining in *Kit^W-sh/W-sh^* mice, we used the recently described ICC marker Anoctamin-1 (Ano1) [28]. In the jejunum of *WT* mice, Ano1 co-stained with Kit specifically the networks of ICCs located at the deep muscular and at the myenteric plexus regions (Figure 4A). By contrast, in the small bowel of *Kit^W-sh/W-sh^* mutant mice, Ano1 immunoreactivity was found exclusively at the level of the deep muscular plexus region (Figure 4B), where only few cells with typical ICC morphology were found (Figure 4B). Our results confirm a malformation of the ICC networks in the *Kit^W-sh/W-sh^* mutant mice (Figure 4A and B). This alteration in the number and distribution of the ICCs in the *Kit^W-sh/W-sh^* mutant mice was associated with significant gut dysmotility found in both naïve mice and mice subjected to laparotomy (Figure 4C and D). Therefore, we conclude that *Kit^W-sh/W-sh^* is not an adequate mouse model to study the role of mast cells in POI.

### Mast cell-deficient *Cpa3^Cre/+^* mice had normal ICC network and regular gastrointestinal transit

**Figure 5:**
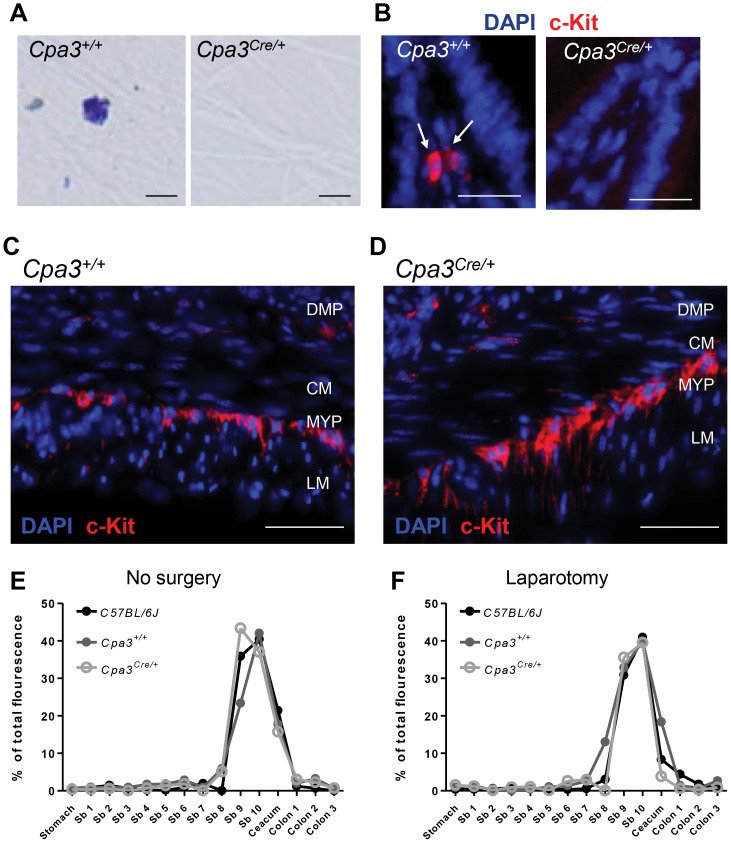
*Cpa3^Cre/+^* mice lack mesenteric and mucosal mast cells but have normal ICC network and gut motility. Naïve *Cpa3^Cre/+^* and littermates control *Cpa3^+/+^* mice were used to analyze intestinal mast cells, ICCs and GI transit. (A) Mesenteric windows from *Cpa3^+/+^* and *Cpa3^Cre/+^* mice were stained with 0.1% toluidine blue. Scale bar 50 µm. (B) Jejunum mucosa sections from naïve *Cpa3^+/+^* and *Cpa3^Cre/+^* mice were immunolabeled with Kit antibody (red) and counterstained with DAPI (blue). Scale bar 25 µm. White arrows are pointing to mast cells in *Cpa3^+/+^* mice. To reveal ICCs, jejunum sections from *Cpa3^+/+^* (C) and *Cpa3^Cre/+^* (D) mice were immunolabeled with Kit (red) and counterstained with DAPI (blue). DMP, deep muscular plexus; MYP, myenteric plexus; CM, circular muscle layer and LM, longitudinal muscle layer. Scale bar 50 µm. GI transit was evaluated in naïve (E) or animal subjected to laparotomy (F) *WT*, *Cpa3^+/+^* and *Cpa3^Cre/+^* mice by assessing dextran-FITC distribution through the GI tract during 90 min after oral gavage. Data are expressed as means. No significant differences were found between the groups of animals (two-way ANOVA).

Considering that naïve untreated *Kit^W-sh/W-sh^* mice have already severe alteration of the gut motility we decided to utilize in our study a newly described mast cell–deficient mouse strain *Cpa3^Cre/+^* with non-perturbed Kit functions [18]. Using toluidine staining and Kit immunolabelling we observed a lack of mast cells both in the peritoneal cavity (Figure 5A) and in the intestinal mucosa (Figure 5B). Interestingly, and contrast to previously described Kit mast cell-deficient mice, Kit labeling of the muscularis externa revealed the presence of a normal network of ICCs in the deep muscular plexus and in the myenteric regions in *Cpa3^Cre/+^* (Figure 5D), with no relievable differences compared to littermate control *Cpa3^+/+^* mice (Figure 5C). To assess whether the presence of normal ICCs in the absence of mast cells is associated with normal gut motility, we performed gastrointestinal transit analyses in *Cpa3^Cre/+^* mice without surgery or only after laparotomy. As depicted in Figure 5E and F, *Cpa3^Cre/+^* mice have normal GI transit with no significant difference when compared to *Cpa3^+/+^* littermate or to *WT* mice. In addition, as shown in Figure S1 the distribution and number of F4/80-positive muscularis externa resident macrophages are comparable in both *Cpa3^Cre/+^* and littermate control mice.

### Mast cell-deficient *Cpa3^Cre/+^* mice develop postoperative ileus and surgery-induced muscularis externa inflammation

**Figure 6:**
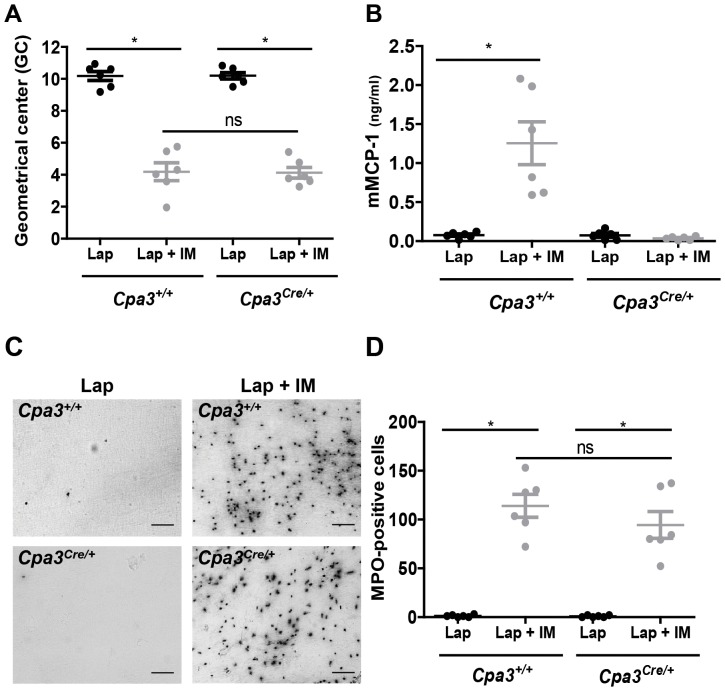
Intestinal manipulation induces postoperative ileus and recruitment of MPO-positive cells in the muscularis externa independently of mast cells. *Cpa3^Cre/+^* and littermates control *Cpa3^+/+^* mice were subjected to laparotomy alone (lap) or to laparotomy plus IM (lap + IM). GI transit was evaluated 24 h after surgery by assessing dextran-FITC distribution through the gastrointestinal tract 90 min after oral gavage. (A) Graph represents GC values. (B) Peritoneal levels of mMCP-1 were determined by ELISA in *Cpa3^+/+^* and *Cpa3^Cre/+^* mice. (C) Representative images of MPO-positive cells in the muscularis externa 24 h after surgery in *Cpa3^Cre/+^* and littermates control *Cpa3^+/+^* mice. (D) Histogram represents numbers of MPO-positive cells in the muscularis externa 24 h after surgery in *Cpa3^Cre/+^* and littermates control *Cpa3^+/+^*. Data expressed as mean ± SEM. * P<0.01 (one-way ANOVA followed by Bonferroni post-hoc test). Dots represent individual mice.

**Figure 7:**
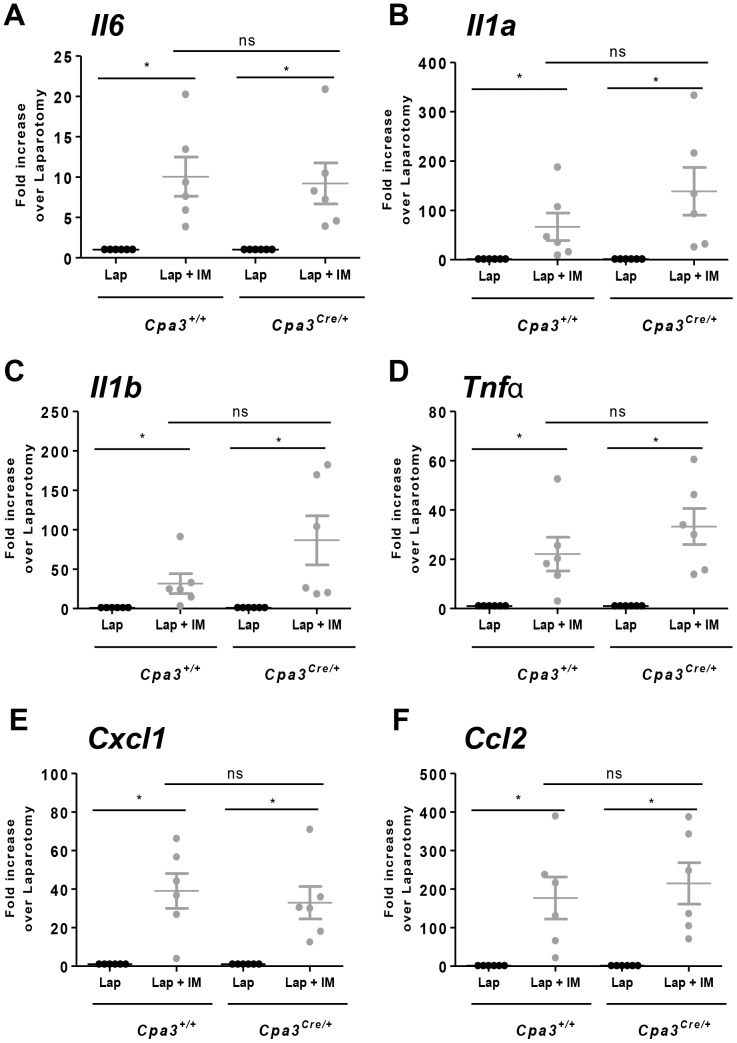
Intestinal manipulation induces muscularis externa inflammation independently of mast cells. *Cpa3^Cre/+^* and littermate control *Cpa3^+/+^* mice were subjected to laparotomy alone (Lap) or to laparotomy plus IM (Lap + IM). Twenty four hours after surgery muscularis externa was collected and cytokines mRNA expression assessed by qPCR. *Il6* (A), *Il1a* (B), *Il1b* (C), *Tnfa* (D), *Cxcl1* (E) and *Ccl2* (F) mRNA expression was evaluated in the jejunum muscularis externa after 24 h. Data expressed as mean ± SEM. * P<0.01 (one-way ANOVA followed by Bonferroni post-hoc test). Dots represent individual mice.

To define if the presence of mast cells has an influence on the development of POI we performed intestinal manipulation in mast cell-deficient *Cpa3^Cre/+^* mice and in *Cpa3^+/+^* littermate controls. Despite the absence of mesenteric as well as intestinal mast cells (Figure 5A and B), IM in *Cpa3^Cre/+^* mice induced a delay in GI transit as shown by a reduction in GC value (*Cpa3^Cre/+^* lap; GC: 10.2±0.2 vs *Cpa3^Cre/+^* IM, GC: 4.1±0.3). The extent of this delay was indistinguishable to littermate control *Cpa3^+/+^* mice (*Cpa3^+/+^* lap; GC: 10.2±0.3 vs *Cpa3^+/+^* IM, GC: 4.2±0.6) (Figure 6A). Next, peritoneal levels of mMCP-1 were assessed in *Cpa3^Cre/+^* and *Cpa3^+/+^* mice 30 minutes after IM. Consistent with their mast cell deficiency, IM did not increase peritoneal mMCP-1 in *Cpa3^Cre/+^* mice (Lap; 0.074±0.0023 vs Lap + IM; 0.036±0.008 ng/ml; Figure 6B) while *Cpa3^+/+^* had a significant increase of this protein in IM versus laparotomy (Lap; 0.076±0.0015 vs Lap + IM; 1.251±0.273 ng/ml; Figure 6B). Intestinal manipulation in mast cell-deficient *Cpa3^Cre/+^* mice also led to inflammatory response in the muscularis externa based on influx of MPO-positive cells (*Cpa3^Cre/+^*; 94±14 number of cells per field; Figure 6C) with no significant difference to littermate controls (*Cpa3^+/+^*; 113±11 number of cells per field; Figure 6C). A normal inflammatory response to IM in the absence of mast cells was also evident by the fact that IM-induced mRNA levels for a range of inflammatory cytokines (*Il6*, *Il1a*, *Il1b*, *Tnfa*, *Cxcl1* and *Ccl2*) in the muscularis externa were comparable in *Cpa3^Cre/+^* mice and littermate controls (Figure 7).

**Figure 8:**
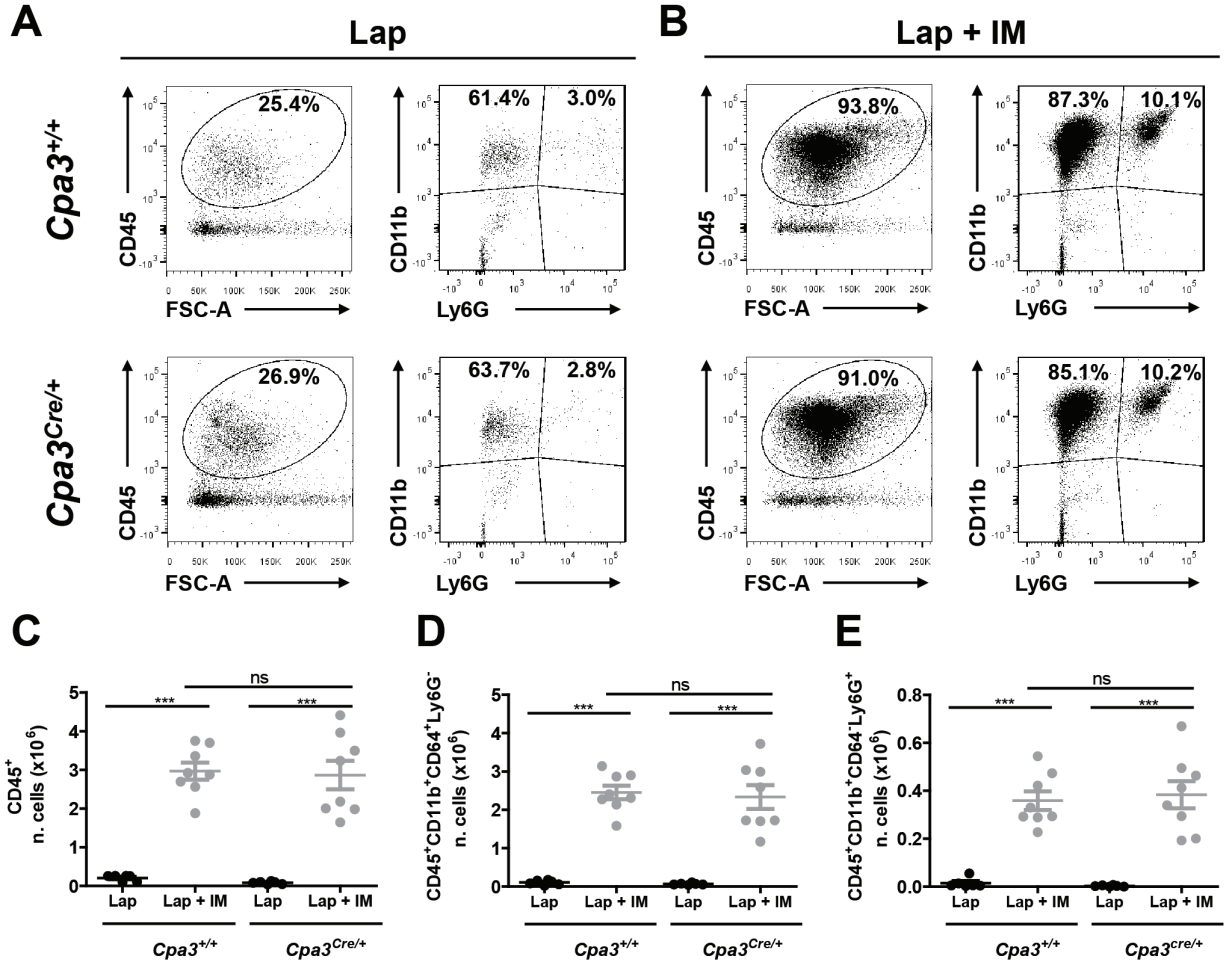
Intestinal manipulation induces recruitment of immune cells in the muscularis externa in the absence of mast cells. *Cpa3^Cre/+^* and littermate control *Cpa3^+/+^* mice were subjected to laparotomy alone (Lap) or to laparotomy plus intestinal manipulation (Lap + IM) and immune cells recruitment in the muscle layer of the small intestine was assessed by flow cytometry. Typical dots plot showing different population of CD45-positive cells in *Cpa3^Cre/+^* and littermate control *Cpa3^+/+^* mice after (A) laparotomy or (B) laparotomy plus intestinal manipulation. Absolute number of CD45 positive immune cells (C), monocytes (D) and neutrophils (E) were calculated from flow cytometry frequencies using viable cell counts. Data expressed as mean ± SEM. *** P<0.001 (one-way ANOVA followed by Bonferroni post-hoc test). Dots represent individual mice.

As previously reported intestinal manipulation leads to the recruitment of neutrophils and monocytes in the muscularis externa. To determine if the lack of mast cells would affect this process, we assessed by flow cytometry the immune cells infiltrating the small bowel muscularis of *Cpa3^Cre/+^* and *Cpa3^+/+^* mice 24 hours after laparotomy or laparotomy plus intestinal manipulation. As evident in Figure 8, no difference in the percentage and in the absolute number of CD45-positive immune cells, monocytes and neutrophils were found between *Cpa3^Cre/+^* and littermate control *Cpa3^+/+^* mice subjected to laparotomy or laparotomy plus intestinal manipulation.

### Cromolyn treatment inhibits mast cell degranulation but does not prevent POI

The use of mast cell stabilizers is an additional strategy to address the roles of mast cells under pathological conditions. Thus, we tested the ability of the a typical mast cell stabilizer, cromolyn, to influence the response to IM [29]. Cromolyn significantly inhibited the release of mMCP-1 in the peritoneal cavity evoked by IM (Figure S2A). Nevertheless, mice treated with cromolyn still developed delayed GI transit following IM, similar to the vehicle treated mice (Figure S2B).

## Discussion

Recent evidence shows that postoperative ileus is mediated by infiltration of leukocytes in the intestinal muscle layer in response to surgical handling of the gut [8]–[10]. Activation of resident macrophages and mast cells has been proposed to be involved in this inflammatory response. However, we here demonstrate, using the mast cell-deficient *Cpa3^Cre/+^* mouse strain, that mast cells do not have a crucial role in the pathogenesis of POI. In the current experiments we used a new mouse strain devoid of both mucosal and connective tissue subtypes of mast cells [18]. In this mouse strain, Cre recombinase is driven by the *Cpa3* locus which is expressed in mast cells from their progenitor stage onwards. *Cpa3^Cre/+^* mice have been used previously to re-address the proposed roles of mast cells in autoimmunity, refuting an involvement of mast cells in the tested models of antibody and T cell-mediated autoimmunity [18].

Considering that Kit signaling is crucial for the development of normal ICC networks and intestinal motility, an important read-out in our POI model, it was critical to use mice with normal ICCs. Indeed, *Cpa3^Cre/+^* mice, in contrast to previously used mast cell-deficient strains, have intact Kit signaling. Consequently, we found normal ICC networks and regular intestinal transit time in *Cpa3^Cre/+^* mice (Figure 5). Moreover, the immune system is not compromised (with the exception of a lower number of basophils) in *Cpa3^Cre/+^* mice, whereas other Kit mutant mast cell-deficient strains have deficiencies in several immune cell subtypes or their functions. Clearly, although no mast cells could be demonstrated based on histology and mast cell mediator release following surgery, *Cpa3^Cre/+^* developed IM-induced intestinal inflammation and delay of gastrointestinal transit, the two hallmark features of POI, to the same extent as *Cpa3^+/+^* littermate mice (Figure 6). The fact that *Cpa3^Cre/+^* mice developed full POI strongly argues against mast cells as crucial player in the development of POI.

In contrast to our new data, a role for mast cells in POI had been invoked based on previous findings. First, mast cell products such as tryptase were released in the peritoneal cavity after intestinal manipulation both in rodents and human [9], [10]. However, in addition to mast cells other immune cells such as basophils and neutrophils may also be a source of tryptase [30]. Second, the mast cell secretagogue compound 48/80 (C48/80) was used to provoke mast cell activation leading to muscularis inflammation, as indicated by an increase in MPO-positive cell infiltration. However, an effect on gut motility was not assessed under these conditions [9]. Moreover, C48/80 has other non-specific effects amongst which inducing oxidative stress [31],vasodilatation [32], and it acts on other immune and non-immune cells including basophils [33], neurons [34], [35] and fibroblasts [36]. In line with these findings, the interpretation of the data acquired using the mast cell stabilizers such as ketotifen or doxantrazole should be used with caution, considering their broad anti-inflammatory effect mainly independent of mast cells [37]–[40]. In previous studies reconstitution experiments with wild type mast cells were used to restore the phenotype which suggested a role for mast cells in POI. Accumulating evidences, however, question this approach. It is now clear that although bone marrow-derived mast cells (BMMCs) can adopt the phenotype of normal tissue mast cells after *in vivo* transfer, numbers, distribution, and functional responses of reconstituted mast cells may not always be physiological [19]. Indeed, several reports using BMMC reconstitution showed a reversal of the phenotype in Kit mutant mice but this has not been confirmed when tested independently in mast cell-deficient mice wild type for Kit. Hence, mast cell reconstitution of Kit mutants does not necessarily reflect mast cell functions in the presence of intact Kit signaling, and may lead to misinterpretation of experimental data and incorrect conclusions [19]. Collectively, none of these previous experiments directly and conclusively proved mast cells to be involved in intestinal pathology.

In addition to these pharmacological studies, the role of mast cells in POI has been traditionally investigated using mice that are mast cell-deficient due to impaired Kit signaling, such as *Kit^W/Wv^* and *Kit^W-sh/W-sh^* mice. However, these mouse strains have pleiotropic phenotypes affecting multiple cell types which comprise normal tissue functions inside and outside of the immune system, as reviewed in [19]. For example, *Kit^W/Wv^* mice suffer from anemia and neutropenia, and lack subsets of intra-epithelial lymphocytes. A strain more recently used in mast cell research, *Kit^W-sh/W-sh^* mice, displays a milder spectrum of abnormalities, but is also affected by immunological abnormalities such as splenomegaly, accumulation of granulocytic myeloid-derived suppressor cells (G-MDSC) [12], expanded myeloid and megakaryocyte populations, neutrophilia and thrombocytosis [13], [14]. These defects in *Kit^W-sh/W-sh^* mice may have contributed to the reported lower inflammatory response, and the reduced number of MPO-positive cells in the muscularis externa after intestinal manipulation [9]. However, here we provide evidence that in *Kit^W-sh/W-sh^*intestinal manipulation induced muscularis externa inflammation (increase in cytokine expression and recruitment of monocytes and neutrophils) even in the absence of mast cells (Figure 1 to 3).

Given that ICC networks in the intestinal wall fail to develop normally in Kit mutant mice [15]–[17], it is not surprising to observe a significant delay in the gastrointestinal transit in naïve *Kit^W-sh/W-sh^* mice (Figure 4). As gastrointestinal transit is one of the major parameters of POI, the ICCs defect should exclude Kit-based mouse models for studying the pathogenesis of ileus. In contrast to Kit mutants, *Cpa3^Cre/+^* mice have intact ICC network and normal intestinal motility (Figure 5) and their immune system is not compromised [18]. Notably, mast cell-deficient *Cpa3^Cre/+^* mice developed IM-induced intestinal inflammation and delay of gastrointestinal transit, the two hallmarks of POI, to the same extent as their mast cell-proficient littermates, indicating that mast cells are not involved in POI (Figure 6 to 8). In support of this conclusion, cromolyn treatment (30 mg/kg; a dose which has been proved to stabilize mast cells in vivo [29]) was ineffective in preventing POI (Figure S2).

In conclusion, our study demonstrates that mast cells, at least in mice, do not play a crucial role in the development of POI, and provide a further example of experimental discrepancies comparing mast cell- and Kit double-deficient mutants versus mast cell-deficient mice without defects in Kit signaling as discussed in [19]. These findings should be definitively taken into account when designing new therapeutic strategies to shorten POI.

## Supporting Information

Figure S1 **Cpa3Cre/+ mice lack mesenteric and mucosal mast cells but have normal ICC network and gut motility.** Naïve WT, KitW-sh/W-sh or Cpa3Cre/+ and littermates control Cpa3+/+ mice were used to analyze the network of resident muscularis externa macrophages. Muscularis externa isolated from the jejunum of WT or KitW-sh/W-sh mice (A) or Cpa3Cre/+ and littermates control Cpa3+/+ mice (B) was stained using an anti-F4/80 antibody. Scale bare 50 µm. Network of F4/80 positive macrophages (red) was detected in all the mouse strains analyzed.
(PDF)

Figure S2 **Cromolyn treatment inhibits mast cell degranulation during intestinal manipulation but does not prevent induction of POI.** WT mice treated with 30 mg/Kg of cromolyn or vehicle were subjected to laparotomy alone (Lap) or to laparotomy plus IM (Lap + IM). (A) Peritoneal levels of mMCP-1 were determined by ELISA. (B) GI transit was evaluated 24 h after IM and GC values calculated. Data are expressed as mean ± SEM. * P<0.05 (one-way ANOVA followed by Bonferroni post-hoc test). Dots represent individual mice.
(PDF)

Table S1 **Primers list.**
(PDF)
