# Correction: Mast Cells Play No Role in the Pathogenesis of Postoperative Ileus Induced by Intestinal Manipulation

**DOI:** 10.1371/annotation/99de087d-c3fc-4e9d-9ee9-552b7524f9e1

**Published:** 2014-01-28

**Authors:** Pedro J. Gomez-Pinilla, Giovanna Farro, Martina Di Giovangiulio, Nathalie Stakenborg, Andrea Némethova, Annick de Vries, Adrian Liston, Thorsten B. Feyerabend, Hans-Reimer Rodewald, Guy E. Boeckxstaens, Gianluca Matteoli

The name of the ninth author is spelled incorrectly. The correct name is: Hans-Reimer Rodewald.

