# Supplementary material for: Mast Cells Play No Role in the Pathogenesis of Postoperative Ileus Induced by Intestinal Manipulation
Source: PLoS One. 2014 Jan 9;9(1):e85304. doi: 10.1371/journal.pone.0085304 (PMC3887017; doi:10.1371/journal.pone.0085304)
Supplement: Figure S1 — Cpa3Cre/+ mice lack mesenteric and mucosal mast cells but have normal ICC network and gut motility. Naïve WT, KitW-sh/W-sh or Cpa3Cre/+ and littermates control Cpa3+/+ mice were used to analyze the network of resident muscularis externa macrophages. Muscularis externa isolated from the jejunum of WT or KitW-sh/W-sh mice (A) or Cpa3Cre/+ and littermates control Cpa3+/+ mice (B) was stained using an anti-F4/80 antibody. Scale bare 50 µm. Network of F4/80 positive macrophages (red) was detected in all the mouse strains analyzed. (PDF) [file pone.0085304.s001.pdf]

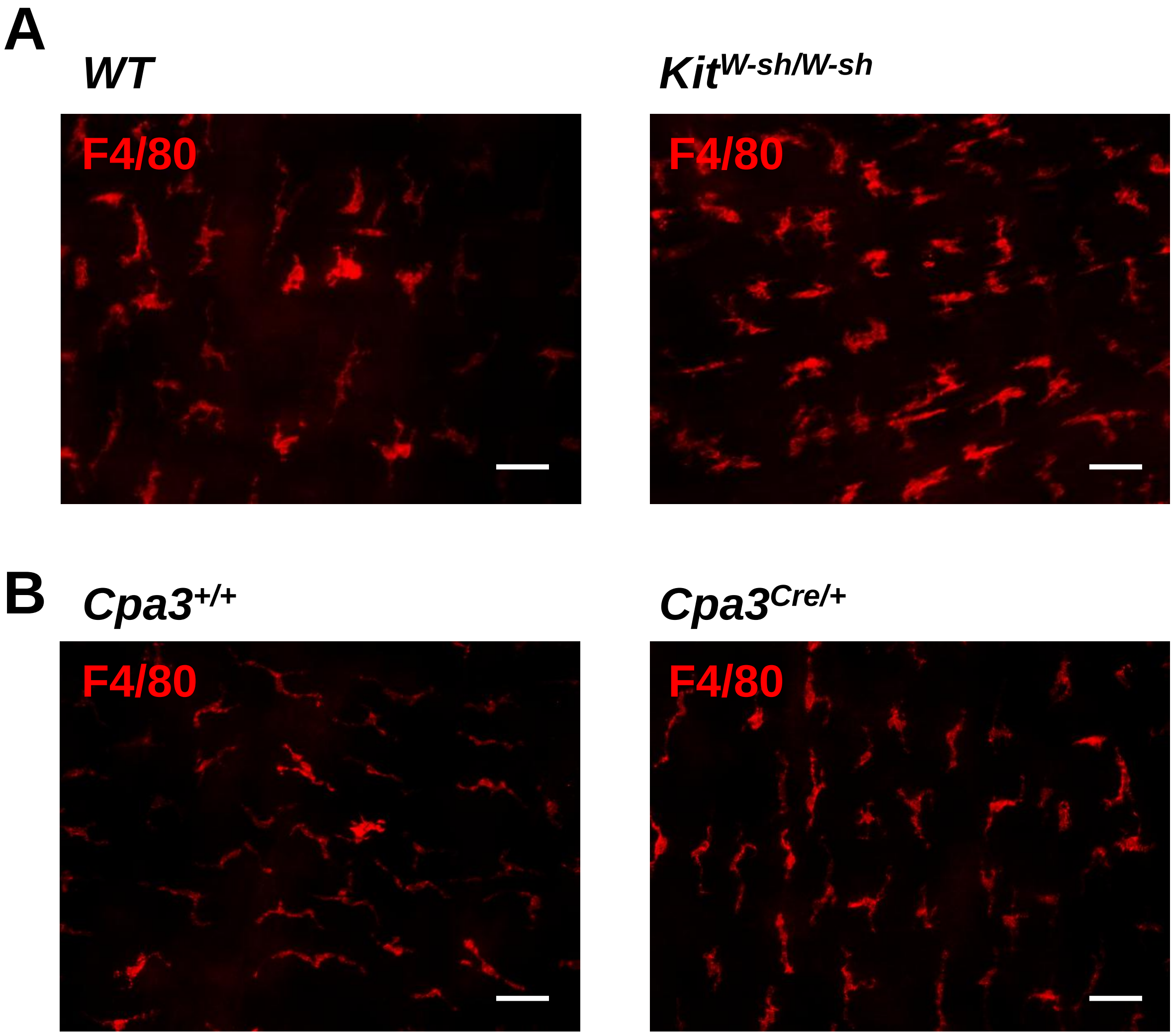

Supporting figure 1. *Cpa3*$^{Cre/+}$ mice lack mesenteric and mucosal mast cells but have normal ICC network and gut motility. Naïve *WT*, *Kit*$^{W\text{-}sh/W\text{-}sh}$ or *Cpa3*$^{Cre/+}$ and littermates control *Cpa3*$^{+/+}$ mice were used to analyze the network of resident muscularis externa macrophages. Muscularis externa isolated from the jejunum of *WT* or *Kit*$^{W\text{-}sh/W\text{-}sh}$ mice **(A)** or *Cpa3*$^{Cre/+}$ and littermates control *Cpa3*$^{+/+}$ mice **(B)** was stained using an anti-F4/80 antibody. Scale bare 50µm. Network of F4/80 positive macrophages (red) was detected in all the mouse strains analyzed.
