# Supplementary material for: Mast Cells Play No Role in the Pathogenesis of Postoperative Ileus Induced by Intestinal Manipulation
Source: PLoS One. 2014 Jan 9;9(1):e85304. doi: 10.1371/journal.pone.0085304 (PMC3887017; doi:10.1371/journal.pone.0085304)
Supplement: Figure S2 — Cromolyn treatment inhibits mast cell degranulation during intestinal manipulation but does not prevent induction of POI. WT mice treated with 30 mg/Kg of cromolyn or vehicle were subjected to laparotomy alone (Lap) or to laparotomy plus IM (Lap + IM). (A) Peritoneal levels of mMCP-1 were determined by ELISA. (B) GI transit was evaluated 24 h after IM and GC values calculated. Data are expressed as mean ± SEM. * P<0.05 (one-way ANOVA followed by Bonferroni post-hoc test). Dots represent individual mice. (PDF) [file pone.0085304.s002.pdf]

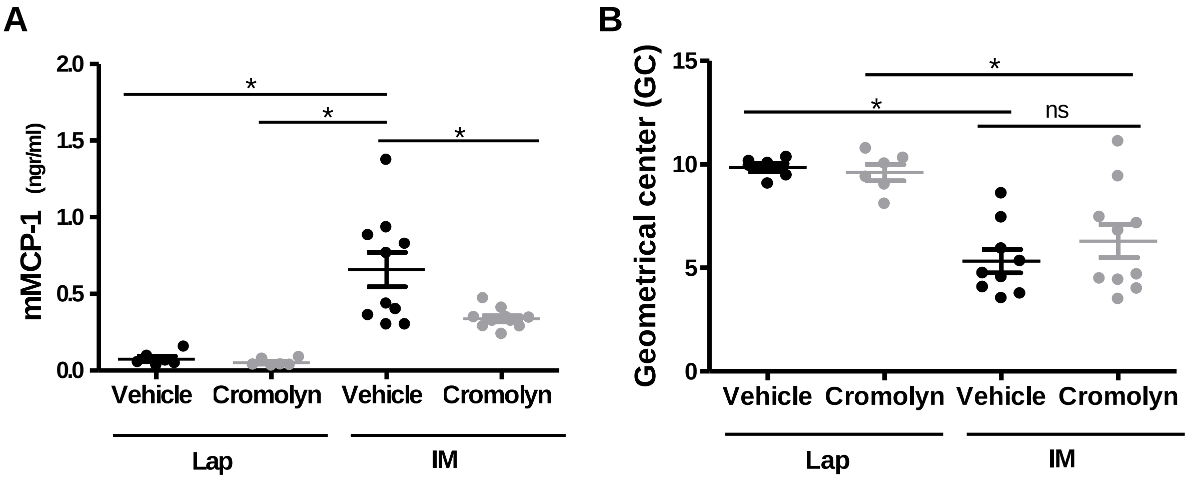

Supporting figure 2. Cromolyn treatment inhibits mast cell degranulation during intestinal manipulation but does not prevent induction of POI. *WT* mice treated with 30 mg/Kg of cromolyn or vehicle were subjected to laparotomy alone (Lap) or to laparotomy plus IM (Lap + IM). (A) Peritoneal levels of mMCP-1 were determined by ELISA. (B) GI transit was evaluated 24 h after IM and GC values calculated. Data are expressed as mean ± SEM. * $P < 0.05$ (one-way ANOVA followed by Bonferroni post-hoc test). Dots represent individual mice.
