# Supplementary material for: Mast Cells Play No Role in the Pathogenesis of Postoperative Ileus Induced by Intestinal Manipulation
Source: PLoS One. 2014 Jan 9;9(1):e85304. doi: 10.1371/journal.pone.0085304 (PMC3887017; doi:10.1371/journal.pone.0085304)
Supplement: Table S1 — Primers list. (PDF) [file pone.0085304.s003.pdf]

Table S1: Primers list

Gene	Sense	Antisense
Rpl32	5'-AAGCGAAACTGGCGGAAAC-3'	5'-TAACCGATGTTGGGCATCAG-3'
Il6	5'-CCATAGCTACCTGGAGTACATG-3'	5'-TGGAAATTGGGGTAGGAAGGAC-3'
Il1α	5'-GAGAGCCGGGTGACAGTATC-3'	5'-ACTTCTGCCTGACGAGCTTC-3'
Il1β	5'-GACCTTCCAGGATGAGGACA-3'	5'-TCCATTGAGGTGGAGAGCTT-3'
Tnfa	5'-TCTTCTCATTCCTGCTTGTGG-3'	5'-CACTTGGTGGTTTGCTACGA-3'
Cxcl1	5'-GCTGGGATTCACCTCAAGAA-3'	5'-TCTCCGTTACTTGGGGACAC-3'
Ccl2	5'-CACGTGTTGGCTCAGCCAGATGC-3'	5'-CCTTCTTGGGGTCAGCACAGACC-3'
